# Supplementary material for: Dual and Opposite Costimulatory Targeting with a Novel Human Fusion Recombinant Protein Effectively Prevents Renal Warm Ischemia Reperfusion Injury and Allograft Rejection in Murine Models
Source: Int J Mol Sci. 2021 Jan 26;22(3):1216. doi: 10.3390/ijms22031216 (PMC7865252; doi:10.3390/ijms22031216)
Supplement: Supplementary file 1 [file ijms-22-01216-s001.pdf]

International Journal of *Molecular Sciences*

MDPI

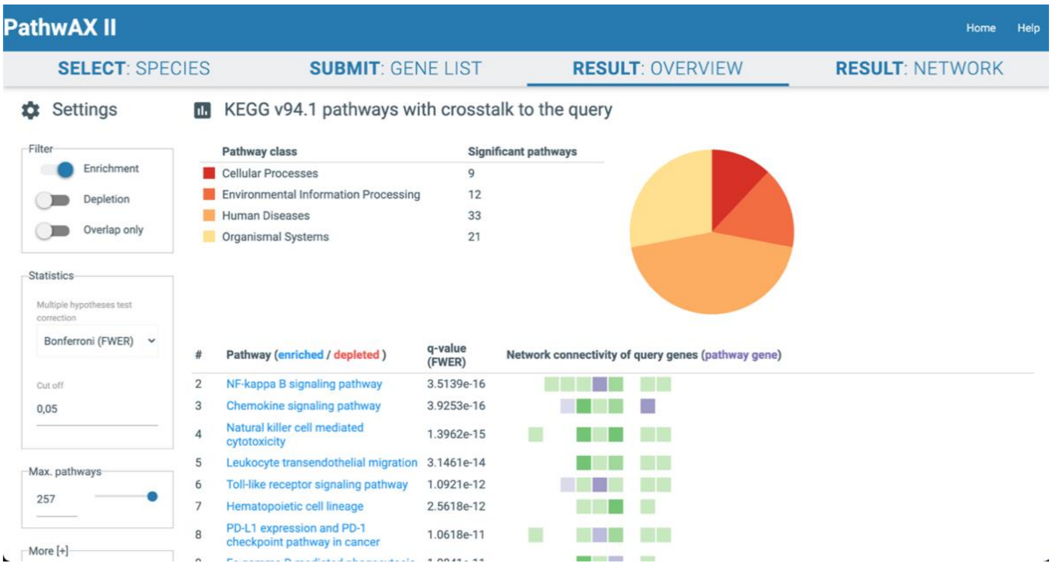

Supplementary Figure S1. PathwAX results showing the modulated pathways with the HYBRI treatment according to the gene array study. The top table and pie diagram summarize the significant pathways and its relation and distribution in different disease and processes. The table below shows the list of all enriched (blue) and depleted (red) pathways related to the genes entered in the database, sorted by increasing FDR (only top part shown). The pathways can also be restricted to a class by clicking on the top table. The network connectivity between the query genes and each pathway is represented in the matrix below. Each colored box represents a gene and mouseover shows the number of links to the pathway. The query genes linked to the pathway are represented by the green boxes and the genes that are part of the pathway are represented by the purple boxes. The greater the connectivity, the darker the tones of the boxes.

Supplementary Table S1. List of all the pathways related with the studied genes introduced to PathwAX database.

Copyright: © 2021 by the authors. Licensee MDPI, Basel, Switzerland. This article is an open access article distributed under the terms and conditions of the Creative Commons Attribution (CC BY) license (http://creativecommons.org/licenses/by/4.0/).

Pathway	PathwaySize	CrossTalk	type	pValue	FWER
**NF-kappa B signaling pathway**	103	26	enrichment	1.37e-18	3.51e-16
**Chemokine signaling pathway**	180	34	enrichment	1.53e-18	3.93e-16
**Natural killer cell mediated cytotoxicity**	94	27	enrichment	5.43e-18	1.40e-15
**Leukocyte transendothelial migration**	115	34	enrichment	1.22e-16	3.15e-14
**Toll-like receptor signaling pathway**	96	22	enrichment	4.25e-15	1.09e-12
**Hematopoietic cell lineage**	96	16	enrichment	9.97e-15	2.56e-12

**PD-L1 expression and PD-1 checkpoint pathway in cancer**	91	24	enrich-ment	4.13e-14	1.06e-11
**Fc gamma R-mediated phagocytosis**	90	28	enrich-ment	4.22e-14	1.08e-11
**Cell adhesion molecules (CAMs)**	158	17	enrich-ment	2.27e-13	5.82e-11
**Leishmaniasis**	75	22	enrich-ment	5.41e-13	1.39e-10
**Focal adhesion**	198	36	enrich-ment	9.62e-13	2.47e-10
**Osteoclast differentiation**	124	22	enrich-ment	1.14e-12	2.93e-10
**Human cytomegalovirus infection**	239	34	enrich-ment	1.44e-12	3.71e-10
**B cell receptor signaling pathway**	78	21	enrich-ment	2.60e-12	6.69e-10
**Rap1 signaling pathway**	215	32	enrich-ment	3.08e-12	7.92e-10
**Axon guidance**	180	26	enrich-ment	4.49e-12	1.15e-9
**Primary immunodeficiency**	40	11	enrich-ment	6.43e-12	1.65e-9
**Tuberculosis**	179	31	enrich-ment	9.75e-12	2.51e-9
**Proteoglycans in cancer**	206	37	enrich-ment	9.90e-12	2.54e-9
**Regulation of actin cytoskeleton**	121	20	enrich-ment	1.37e-11	3.51e-9
**Th1 and Th2 cell differentiation**	92	17	enrich-ment	3.71e-11	9.53e-9
**T cell receptor signaling pathway**	108	21	enrich-ment	1.07e-10	2.75e-8
**Yersinia infection**	127	26	enrich-ment	1.92e-10	4.92e-8
**Kaposi sarcoma-associated herpesvirus infection**	193	27	enrich-ment	2.08e-10	5.33e-8
**Toxoplasmosis**	116	23	enrich-ment	3.90e-10	1.00e-7
**Cytokine-cytokine receptor interaction**	266	12	enrich-ment	6.10e-10	1.57e-7
**Salmonella infection**	79	25	enrich-ment	6.17e-10	1.58e-7
**MAPK signaling pathway**	298	28	enrich-ment	1.08e-9	2.77e-7
**Pertussis**	72	20	enrich-ment	1.11e-9	2.85e-7
**Human immunodeficiency virus 1 infection**	227	32	enrich-ment	1.27e-9	3.25e-7
**JAK-STAT signaling pathway**	153	14	enrich-ment	8.16e-9	0.00000210

**Hepatitis B**	160	25	enrich-ment	1.26e-8	0.00000323
**Th17 cell differentiation**	110	21	enrich-ment	1.77e-8	0.00000456
**Viral protein interaction with cytokine and cytokine receptor**	83	8	enrich-ment	1.84e-8	0.00000472
**Staphylococcus aureus infection**	99	11	enrich-ment	1.99e-8	0.00000512
**Platelet activation**	129	24	enrich-ment	2.10e-8	0.00000539
**Adherens junction**	75	22	enrich-ment	4.14e-8	0.0000106
**Herpes simplex virus 1 infection**	336	25	enrich-ment	4.28e-8	0.0000110
**Necroptosis**	154	33	enrich-ment	1.04e-7	0.0000268
**Phospholipase D signaling pathway**	152	18	enrich-ment	2.23e-7	0.0000573
**Bacterial invasion of epithelial cells**	77	22	enrich-ment	3.55e-7	0.0000912
**Ras signaling pathway**	238	22	enrich-ment	3.75e-7	0.0000963
**Fc epsilon RI signaling pathway**	71	14	enrich-ment	6.81e-7	0.000175
**Epstein-Barr virus infection**	222	30	enrich-ment	7.67e-7	0.000197
**Systemic lupus erythematosus**	112	25	enrich-ment	0.00000134	0.000345
**Apoptosis**	137	24	enrich-ment	0.00000198	0.000509
**Complement and coagulation cascades**	86	10	enrich-ment	0.00000229	0.000590
**Chagas disease (American trypanosomiasis)**	105	16	enrich-ment	0.00000262	0.000673
**MicroRNAs in cancer**	163	20	enrich-ment	0.00000351	0.000901
**PI3K-Akt signaling pathway**	347	29	enrich-ment	0.00000357	0.000919
**Measles**	147	20	enrich-ment	0.00000504	0.00130
**Viral carcinogenesis**	206	32	enrich-ment	0.00000619	0.00159
**Tight junction**	169	27	enrich-ment	0.00000816	0.00210
**Influenza A**	167	23	enrich-ment	0.00000916	0.00235
**Amoebiasis**	100	13	enrich-ment	0.0000153	0.00393
**Intestinal immune network for IgA production**	48	6	enrich-ment	0.0000177	0.00454

**Hepatitis C**	158	21	enrich-ment	0.0000179	0.00461
**Growth hormone synthesis, secretion and action**	119	14	enrich-ment	0.0000267	0.00686
**Phagosome**	171	27	enrich-ment	0.0000269	0.00692
**Pancreatic cancer**	76	13	enrich-ment	0.0000297	0.00763
**Relaxin signaling pathway**	132	14	enrich-ment	0.0000462	0.0119
**Endocytosis**	245	27	enrich-ment	0.0000498	0.0128
**Viral myocarditis**	78	13	enrich-ment	0.0000541	0.0139
**NOD-like receptor signaling pathway**	177	20	enrich-ment	0.0000570	0.0146
**TNF signaling pathway**	116	11	enrich-ment	0.0000649	0.0167
**Acute myeloid leukemia**	69	11	enrich-ment	0.0000727	0.0187
**Choline metabolism in cancer**	97	12	enrich-ment	0.0000749	0.0193
**Signaling pathways regulating pluripotency of stem cells**	140	12	enrich-ment	0.0000818	0.0210
**Hepatocellular carcinoma**	176	18	enrich-ment	0.0000847	0.0218
**Human T cell leukemia virus 1 infection**	233	23	enrich-ment	0.000104	0.0268
**Estrogen signaling pathway**	133	20	enrich-ment	0.000105	0.0269
**Malaria**	53	6	enrich-ment	0.000113	0.0291
**Pathways in cancer**	168	12	enrich-ment	0.000137	0.0352
**Neurotrophin signaling pathway**	121	16	enrich-ment	0.000154	0.0396
**Sphingolipid signaling pathway**	124	15	enrich-ment	0.000182	0.0468
